# Post-traumatic Stress Symptoms and Serotonin Transporter (5-HTTLPR) Polymorphism in Breast Cancer Patients

**DOI:** 10.3389/fpsyt.2021.632596

**Published:** 2021-04-21

**Authors:** Luigi Zerbinati, Martino Belvederi Murri, Rosangela Caruso, Maria Giulia Nanni, Wendy Lam, Silvia De Padova, Silvana Sabato, Tatiana Bertelli, Giulia Schillani, Tullio Giraldi, Richard Fielding, Luigi Grassi

**Affiliations:** ^1^Department of Biomedical and Specialty Surgical Sciences, Institute of Psychiatry, University of Ferrara, Ferrara, Italy; ^2^University Hospital Psychiatry Unit, Integrated Department of Mental Health and Addictive Behavior, University S. Anna Hospital and Health Trust, Ferrara, Italy; ^3^Centre for Psycho-Oncological Research and Training, School of Public Health, The University of Hong Kong, Pok Fu Lam, Hong Kong; ^4^Psycho-Oncology Unit, Istituto Scientifico Romagnolo per lo Studio e la Cura dei Tumori (IRST) Istituto di Ricovero e Cura a Carattere Scientifico (IRCCS), Meldola, Italy; ^5^Child Onco-Hematology Unit, Maternal and Child Health, Istituto di Ricovero e Cura a Carattere Scientifico (IRCSS) Burlo Garofolo, Trieste, Italy; ^6^Section of Pharmacology, Department of Life Sciences, University of Trieste, Trieste, Italy

**Keywords:** 5-HTTLPR polymorphism, breast cancer, post-traumatic stress symptoms, post-traumatic stress disorder, serotonine transporter

## Abstract

**Introduction:** Post-traumatic Symptoms (PTSS) and Post-traumatic Stress Disorder (PTSD) have been reported to affect a quite significant proportion of cancer patients. No study has examined the relationship between serotonin transporter gene-linked polymorphic region (5-HTTLPR) and cancer, including Gene-Environment interactions between this polymorphism and specific causes of distress, such as cancer related problems (CRP) or life stressful events (SLE).

**Methods:** One hundred and forty five breast cancer outpatients participated in the study and were assessed using the Impact of Event Scale (IES), the Problem List (PL) developed by the National Comprehensive Cancer Network (NCCN) Distress Management Guidelines and the Paykel's Life Events Interview to evaluate the exposure to SLE during the year before the cancer diagnosis. Each patient was genotyped for 5-HTTLPR polymorphism by analyzing genomic DNA obtained from whole blood cells. Gene-Environment interactions were tested through moderation analysis.

**Results:** Twenty-six patients (17.7%) were classified as PTSS cases using the IES. Genotype and phenotype distributions did not differ across individuals with/without PTSS (genotype: χ^2^ = 1.5; df = 2; *p* = 0.3; phenotype χ^2^ = 0.9; df = 1; *p* = 0.2). For both the genotype and phenotype model, using CRP as a predictor showed significant gene-environment interactions with IES total score (*p* = 0.020 and *p* = 0.004, respectively), with individuals carrying the *l/l* allele showing a greater probability of experiencing PTSS. No interaction was found in relationship to SLE (*p* = 0.750).

**Conclusion:** This study showed a significant GEI between CRP and PTSS in breast cancer patients, with carriers of the *l/l* allele showing indicators consistent with greater sensitivity to stress.

## Introduction

Cancer is a stressful traumatic experience associated with a high prevelance of adjustment and stress-related disorders (1). Data show that up to 30–35% of cancer patients develop Post-traumatic Stress Symptoms (PTSS), sub-syndromal or even full-fledged Post-traumatic Stress Disorder (PTSD) at some point of the disease trajectory (2–4), including breast cancer (5, 6). PTSD in cancer patients can negatively impact the quality of life, cognitive, social, work-related, and physical functioning and the treatment process (7, 8), thus potentially affecting the course of the disease.

Literature on survivors of life-threatening events, such as natural disasters, wars or severe childhood abuse, have shown that the risk of developing trauma-related psychopathology can be modulated by individual genetic background (9). Variants of the 5-hydroxy-tryptamine (5HT) transporter gene (SLC6A4) have been studied for their role in the vulnerability to Post-traumatic Stress Disorder (PTSD) among individuals exposed to environmental stress (10). In particular, the 5HT-transporter gene-promoter variant (5HTLPPR), consisting in a long (*l*) and a short (*s*) variant, has been identified as an effect modifier (moderator) of the association between traumatic events and the onset of PTSD or post-traumatic stress symptoms (PTSS). Individuals homozygous for the short allele (*s/s*) of the 5-HTTLPR display a higher likelihood of developing PTSD after exposure to significant traumatic events, compared with *s/l* or *l/l* carriers (11). A recent meta-analysis pooled the results of 14 studies, of which five had used a longitudinal design, concluding that 5HTTLPR genotype was a significant moderator of the association between stress and PTSD, with a significant increment in PTSD incidence/prevalence associated with the presence of the *s* allele (9). Also, carriers of *s* alleles displayed more anxious arousal and symptom re-experiencing than *l* carriers, but not symptoms of avoidance, numbing, or dysphoric arousal (12). However, some inconsistent findings were reported by other studies, either detecting a higher prevalence of PTSD among *l/l* than among *s/s* and *s/l* carriers (13, 14), showing additive interaction between the *l/l* genotype and traumatic events to determine PTSD (15) or failing to find an association between stress and 5HTTLPR genotype in PTSD.

Most of the cited studies, however, only examined the presence of main effects and did not examine additive interactions (16, 17). Results of Gene-Environment Interaction (GEI) studies are not entirely concordant, and more research is needed to understand the relationship between stress and 5HTTLPR genotype (9, 18). Variability of findings may derive from clinical characteristics, as well as differences in the definitions of stress or psychopathology. Also, and more importantly, the 5-HTTLPR polymorphisms and its possible role in mental health problems secondary to cancer have only been reported in a few studies of cancer patients examining the association between 5-HTTLPR polymorphisms and depression in cancer, which have returned contradictory results (19–22).

Thus, the lack of data on the interactions between the adaptation of cancer patients and 5-HTTLPR polymorphisms, specifically regarding the development of PTSS arising from stress consequent to cancer-related problems or life events prompted us to explore this area. Specifically, we examined if interactions between 5-HTTLPR genotype and reported environmental stress predicted PTSS among breast cancer patients, with the hypothesis that patients with different 5HTTLPR genotypes would report different levels of cancer-related stress and PTSS.

## Materials and Methods

### Participants

A convenience sample of breast cancer patients (*n* = 145) were enrolled at the out-patient and day-hospital clinics of the Unit of Medical Oncology, University S. Anna Hospital in Ferrara. Criteria for recruitment were: (i) diagnosis of cancer in the previous 6 months; (ii) a Karnofsky Performance Status scale >80; (iii) absence of cognitive deficits or involvement of the CNS at the clinical evaluation; (iv) age between 18 and 70 years. All the participants completed a comprehensive psychosocial assessment conducted by a researcher with extensive experience in psycho-oncology and blood sample was collected in order to characterize 5-HTTLPR polymorphism. The study was conducted after having been approved by the relevant institutional Ethical Committee, and having received informed consent by each participant. The original contributions presented in the study are publicly available. This data can be at the following link: https://figshare.com/articles/dataset/5HTPLRR_PTSS_sav_pdf/13713541.

### Psychosocial Assessment

#### Post-traumatic Stress Symptoms

The 15-item Impact of Event Scale-IES (23) assesses the frequency of intrusive and avoidant cognitions and behaviors, the two main clinical dimensions of PTSD, on a 4-point Likert scale, in response to specific events. Item scores are summed to obtain a total IES Total score (range score of 0–75) or the Intrusion and Avoidance subscale scores (Intrusion subscale: 7 items; score range 0–35; Avoidance subscale: 8 items, score range 0–40). The following cut-off scores are used to differentiate the severity of stress responses on the IES Total score: 0–8, no or subclinical PTSS; 9–25, mild PTSS; 26–43, moderate; ≥44, severe PTSS (24, 25). The IES (IES Total score) is reported to have good validity for detecting patients with a diagnosis of PTSD obtained by clinical interview (26). In particular, a cut-off of total IES score ≥35 had high sensitivity (0.89) and specificity (0.94) for a diagnosis of PTSD obtained with a structured interview in a large validation study of Danish breast cancer patients (27). In this sample, the reliability of the IES was satisfactory (Cronbach's α for Intrusion: 0.82; Avoidance: 0.79; IES total: 0.87) confirming the usefulness of the IES in cancer research (28).

#### Assessment of Environmental Stress

In addition to the experience of cancer, patients may be exposed to other types of environmental stress, including previous life events and problems deriving from the illness. Thus, we deemed it useful to investigate their differential effects by employing two more widely used instruments.

Paykel's Life Events Interview retrospectively documented stressful life events over the 12 months preceding the diagnosis of cancer. The interview assesses the occurrence of 64 possible life stressful events (SLE) related to the work, education, finance, health, bereavement, family, and social domains, using the count of events as an index of environmental stress (29). Given the aims of the study, we did not rate those health problems that could be related to cancer among SLE (e.g., symptoms, diagnostic procedures, or visits that would be later attributed to the illness).

Cancer-related Problems (CRP) were assessed by using the Problem List (PL) developed as a tool by the National Comprehensive Cancer Network (NCCN) Distress Management Guidelines (30, 31). This instrument rates the presence (yes/no) during the prior week of 34 problems related to cancer from a list of five categories: practical, family, emotional, physical problems, and spiritual/religious concerns. The total count of CRP is generally used to guide and facilitate the interview used to screen for distress in cancer patients. However, given the aim of the study, we computed CRP as the count of only practical, social/family, and physical problems, while excluding emotional and spiritual issues due to their expected correlation with the outcome.

### Genotyping

Participants were genotyped for 5-HTTLPR polymorphism by analyzing genomic DNA obtained from whole blood cells, using standard procedures (GenEluteTM blood Genomic DNA Kit, Sigma). Polymerase chain reaction (PCR) amplification of 5-HTTLPR was performed using the primer sequences described by Gelernter et al. (32), the forward primer having the sequence (5′-ATGCCAGCACCTAACCCCTAATGT-3′) and the reverse (5′-GGACCGCAAGGTGGGCGGGA-3′). This amplifies a 419 base pair product for the 16 repeat (“*l*”) allele and a 375 base pair product for the 14 repeat (“*s*”) allele. PCR was carried out on Gene AMP 9700 (PE Applied Biosystems), using the following cycling conditions: initial 10 min denaturing step at 95°C, followed by 40 cycles of 40 s of denaturation at 94°C, 45 s of annealing at 56°C, 40 s of extension at 72°C, and a final extension phase of 72°C for 10 min. Reactions were performed in a final volume of 20 μL product containing 0,8 μL (20 ng) of DNA, 0,8 μL (10 pmol) of each primer, 0,8 μL (10 pmol) of dNTPs, 16,8 μL of GC-RICH PCR System (ROCHE Molecular Biomedicals), containing 8 μL of GC-RICH Resolution Solution, 5 M, 8 μL of GC-RICH reaction buffer (7,5 mM MgCl2 and 1,5 mM DMSO) and 0,8 μL of GC-RICH Enzyme Mix. PCR products were separated on a 2% agarose gel (MultiABgarose, ABgene) supplemented with Ethidium bromide (0.03%) and visualized by ultraviolet transillumination. Genomic data were analyzed according to an additive genotype model (three groups: *s/s* vs. *s/l* vs. *l/l* genotypes) and according to phenotype groups as the dominant model (subjects carrying vs. not carrying the *s* allele). All the genetic analyses, including the identification of and the fragment lengths, were performed in the Pharmacogenetics Lab of Department of Life Sciences, University of Trieste, Trieste, Italy by the researchers of Department of Life Science.

### Statistical Analyses

Between-group differences in psychosocial variables according to 5-HTTLPR polymorphisms were explored using Chi-Square, *t*-test, and Analysis of Variance (ANOVA). Gene-environment interactions were examined by testing whether the 5-HTTLPR polymorphism had a moderating role (effect modifier) on the association between stressful life events and PTSS. Moderation analyses were performed with the PROCESS macro, 3.4 release (33). The macro employs conditional process analysis based on OLS and logistic regression modeling and provides bias-corrected 95% confidence intervals (CIs) using bootstrap calculation (*n* = 1,000 iterations). Separate models were analyzed using IES score (indicating the severity of PTSS) as the dependent variable, CRP (independent variable) as indices of exposure to stressful events and the genotype model (*s/s, s/l, l/l*) as a multicategorical moderator. Similarly, still using IES score as the dependent variable, additional analyses included SLE as a different exposure to stress, and the phenotype model to be tested as a different dichotomous moderator. In order to prevent Type I errors in null hypothesis, given the presence of multiple analyses, the Bejamini Hockberg False Discovery Rate (FDR) correction was used (34). All analyses were conducted using SPSS 21.0 (IBM corporation).

Finally, a *post-hoc* power analysis was conducted using G^*^Power (35).

## Results

Data pertaining to 145 patients were collected. The socio-demographic and clinical characteristics of the sample are shown in Supplementary Table 1. All the patients were female with a mean age of 55.87 ± 8.98; the majority were married (75.8%), with no past history of psychological disorders (65.5%) and 44.8% were employed. Most patients had localized disease (84.8%), with 93 patients (64.1%) undergoing quadrantectomy. Almost half of the patients (48.3%) did not receive any further anticancer intervention, with the remaining subjects receiving chemotherapy (22.7%), hormonal therapy (14.5%), or combined therapy (14.5%).

The frequencies of the three 5-HTTLPR genotypes were: *ll*, 31.3% (*n* = 45); *sl*, 45.5% (*n* = 66); and *ss*, 23.4% (*n* = 34), comparable with previous reported frequencies in similar samples (Hardy–Weinberg equilibrium: χ2 = 0.90416; *p* = ns). The phenotype groups (*s/s* + *s/l*; *l/l*) did not differ by age, disease stage or treatment (Supplementary Table 2). Twenty-six patients (17.7%) were classified as PTSS cases using the IES cut-off score of 35. Genotype and phenotype distributions were comparable, with no significant difference between PTSS cases vs. non-cases (genotype: χ^2^ = 1.5; df = 2; *p* = 0.3; phenotype χ^2^ = 0.9; df = 1; *p* = 0.2).

**Figure 1:**
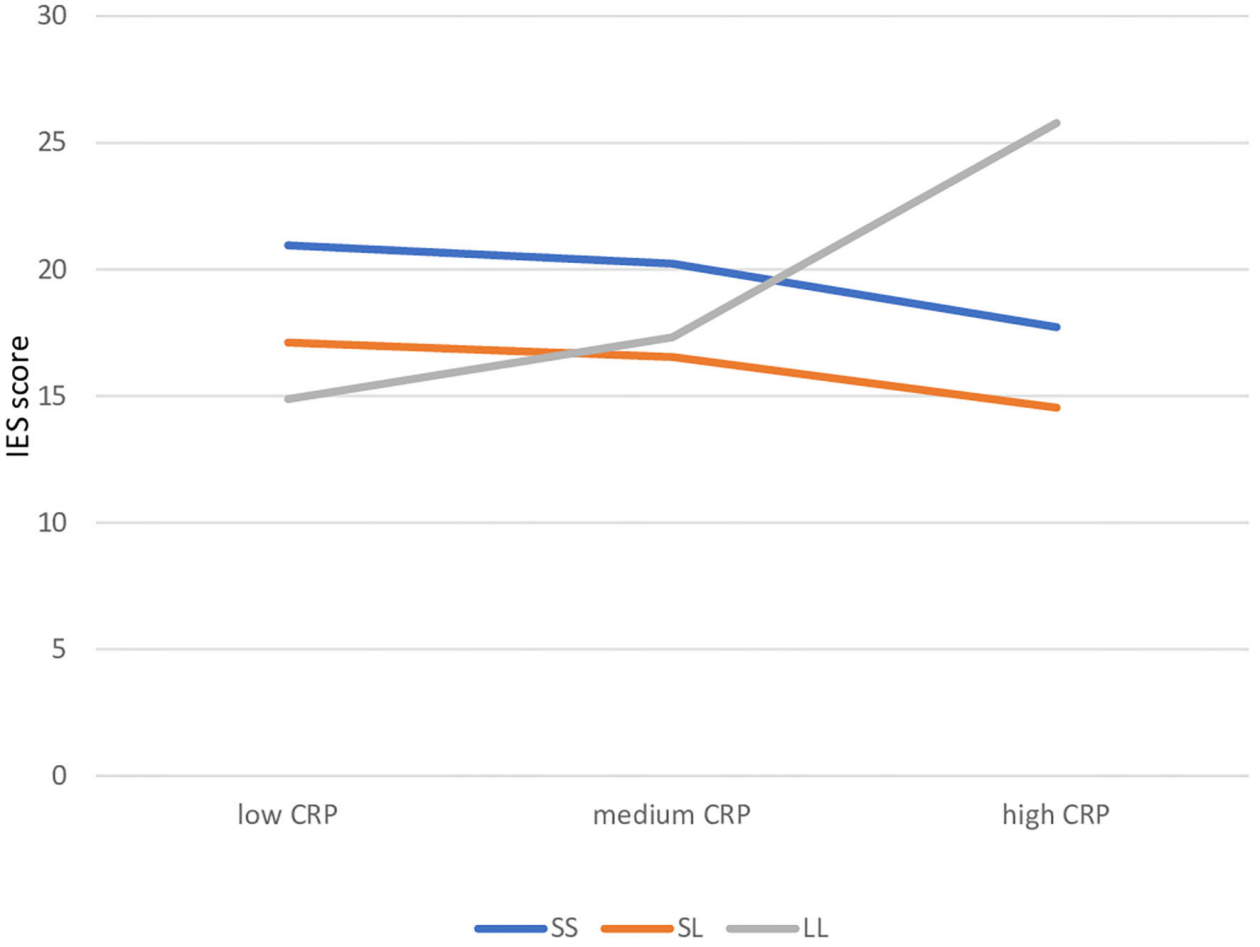
Gene-environment interaction: simple slopes of cancer-related problems in predicting IES score by 5HTTLR genotype. CRP, Cancer Related Problems.

We used SLE or CRP as indices of stress as predictors; SLE did not yield significant gene-environment interactions (*p* = 0.750, Table 1), whereas significant gene × environment interactions were detected with CRP, on IES total score (both in genotype and phenotype models, *p* = 0.020 and *p* = 0.004). In particular, individuals carrying the *l/l* allele displayed a significant, positive association between CRP and IES score (*greater* probability of being a PTSS case), in contrast with those carrying the *s/s* or *s/l* alleles (Table 1 and Supplementary Table 3; interaction between CRP and IES score depicted in Figure 1). Results were similar in further exploratory analyses using the IES subscale Avoidance as the continuous outcomes (*p* = 0.013), but not for the IES subscale Intrusion (*p* = 0.102; Supplementary Table 3). The results did not substantially change after adjusting models for age, previous psychiatric disorders or current depressive symptoms.

**Table 1:** Gene-environment interactions: genotype models.

**Outcome**	**Exposure**	**Moderator**	**Test(s) of highest order unconditional interactions(s):**	**Effects at levels of the moderator**	**Effect**	***se***	***p***	**LLCI**	**ULCI**
**Cancer-related problems**									
IES	CRP	Genotype	R2-chng: 5%, *F*_2,140_ = 4.02, *p* = 0.0200; *q* = 0.0250	*s/s*	−0.72	0.92	0.44	−2.5584	1.11
				*s/l*	−0.57	0.74	0.43	−2.04	0.89
				***l/l***	**2.43**	**0.91**	**<0.01**	**0.62**	**4.23**
**Paykel life events**									
IES	SLE	Genotype	R2-chng: 0.4%, *F*_2,140_ = 0.28, *p* = 0.7503; *q* = 0.05	*-*	-	-	-	-	-

*CRP, Cancer Related Problems; SLE, number of life stress events before diagnosis. Effect, standard error, p value, lower and upper confidence interval*.

## Discussion

This is the first study that, to our knowledge, examined the role of the 5-HTTLPR polymorphism and GEI interaction in PTSS in cancer patients.

No difference was found between groups according to genotype and phenotype distributions as far as PTSS is concerned, consistent at a first look with the negative data obtained in cancer populations as far as depressive disorders are concerned (22). However, when examining the GxE interaction, the *l/l* genotype was associated with a higher probability of PTSS in patients reporting greater cancer-related stress scores, but not those with high prior SLEs. These results contrast with those from Zhao et al. (9) meta-analysis of 14 studies carried out in non-cancer settings, where the *s/s*, rather than the *l/l* genotype, was associated with greater likelihood of PTSD or depression classifications (36). However, our results are in line with other studies showing a higher PTSD case prevalence among *l/l* rather than *s/s*, or no *s/l* allele variant associations with PTSD secondary to trauma (13, 14, 16, 17). Grabe et al. (15) for example detected a significant GEI only when three or more traumatic events (lifetime) were used as the threshold for exposure to environmental stress. Unlike our study, events were not specifically related to physical health, but may have encompassed a more intense and/or chronic type of stress.

Explaning these data is not easy. A tentative explanation may depend on the enduring nature of day-to-day exposure to cancer-related problems (4), specifically in breast cancer (8). Regarding depression but not PTSS, some data showing that among breast cancer patients *l/l* carriers displayed more severe depressive symptoms than other participants, although a proper GEI was not tested (37). Speculatively, it is possible that different interactions can be evident between isoforms of the serotonin transporter and different levels of serotonin released into the bloodstream by breast cancer (38): *s/s* carriers, due to their lower reuptake activity, may thus maintain higher levels of circulating serotonin, hence a reduced risk of developing stress-related psychopathology. Consistently, serotoninergic antidepressant side-effects and effectiveness seem more pronounced among *non-s* carriers (39). Clearly, these findings need to be replicated before postulating whether the mechanisms of PTSS associated with breast, or other types of cancer might be different from those deriving from other life-threatening events (4, 8), including the possible implications in terms of antidepressant treatment (40).

The strength of the study is that it is the first examining the possible role of the 5-HTTLPR polymorphism in PTSS risk among cancer patients. Also, we used a specific tool to assess cancer-related stressful problems (NCCN CRP) that represent the most significant source of stress at different levels (i.e., primary stress represented by the cancer-related problem itself, secondary stress represented by the interpersonal, psychological practical consequences of the primary stress).

There are however significant limitations that should be mentioned. First, genotype and phenotypic stratification within a small sample size of this study limited both power and generalizability of our results. Nonetheless, a *post-hoc* power analysis revealed that using an α error probability of 0.05, our sample of 145 subjects yielded a power of 78% of detecting an Effect Size *f*^2^ = 0.052; this result is very close to the power of 80% that is conventionally accepted as appropriate for most studies. Also the cross-sectional design does not allow to prospectively generalize our results. Second, according to Paykel's interview, stressful events during the life trajectory were retrospectively assessed considering only the previous year (therefore before diagnosis). While this method prevents the detection of whole-life-span negative events (particularly childhood adversity), other studies in non-cancer settings that examined much longer times prior to PTSD, may suffer from inflated SLE reporting due to recall bias (9). Additionally, cancer related PTSS may be the result of different forms of stress. In our analysis we focused on acute stress related to specific CRP during the previous week, but it is likely that at least some patients had been undergoing significant stress for more time, since the diagnosis of cancer was done within the previous 6 months. Accordingly, in both human studies (41, 42) as well as in animal models (43), chronic stress represents a vulnerability for the development of PTSD. The fact that SLE did not show a significant GXE interaction might suggest that this vulnerability, if present, might be cancer specific. To fully address this problem, an analysis taking into account the 5-5HTTLPR system and both CRP during both the previous week as well as during the last months (e.g., moderated moderation) would confirm this hypothesis.

Another limitation lies in the usage of an older version of the IES which, although shown to be reliable for PTSD in cancer patients (28), did not take into account hyperarousal symptoms that characterize these patients and which are fully explored using the newer IES-R (44). Lastly, we did not assess PTSD or other psychiatric comorbidities with a standardized psychiatric interview nor did we investigate other polymorphisms and functional variants of the 5-HTTLPR system (e.g., the L_G_ polymorphism).

## Conclusions

In conclusion, our study detected a gene-environment interaction between cancer-related problems and PTSS in cancer patients, so that carriers of the *l/l* allele in the promoter region of the 5-HTTLPR gene apparently reported greater sensitivity to stress.

More attention and research to key environmental and biological risk factors for PTSD (e.g., childhood adversity, possible biological predisposing factors, including stress-driven activation of biological, and endocrine pathways) that are influential in the maintenance of PTSD and worse patient outcomes (45). With respect to this, further studies are needed to better clarify the role of the serotonin system in the development of PTSS in order to identify women who may be at heightened risk of PTSD amongst breast cancer patients and to properly integrate psychopharmacological and psychological approaches to lessen the negative impact of this chronic mental health condition (46, 47).

## Data Availability Statement

The datasets presented in this study can be found in online repositories. The names of the repository/repositories and accession number(s) can be found below: https://figshare.com/10.6084/m9.figshare.13713541.

## Ethics Statement

The studies involving human participants were reviewed and approved by Central Emilia Wide Area Ethical Committee of the Emilia-Romagna Region. The patients/participants provided their written informed consent to participate in this study.

## Author Contributions

LG, TG, RF, and WL: conceptualization. TB, SD, GS, SS, RC, and MN: investigation. MM and LZ: formal analysis. MM, LZ, and TG: methodology. LZ and MM: data curation and writing—original draft preparation. LG: writing—review and editing. RF, TG, and WL: validation. LG: supervision. All authors have read and agreed to the published version of the manuscript.

## Conflict of Interest

The authors declare that the research was conducted in the absence of any commercial or financial relationships that could be construed as a potential conflict of interest.

## References

[1] Mitchell AJ Chan M Bhatti H Halton M Grassi L Johansen C. Prevalence of depression, anxiety, and adjustment disorder in oncological, haematological, and palliative-care settings: a meta-analysis of 94 interview-based studies. Lancet Oncol. (2011) 12:160–74. 10.1016/S1470-2045(11)70002-X 21251875

[2] Kangas M Henry JL Bryant RA. Posttraumatic stress disorder following cancer: a conceptual and empirical review. Clin Psychol Rev. (2002) 22:499–524. 10.1016/S0272-7358(01)00118-0 12094509

[3] Rustad JK David D Currier MB. Cancer and post-traumatic stress disorder: diagnosis, pathogenesis and treatment considerations. Palliat Support Care. (2012) 10:213–23. 10.1017/S1478951511000897 22436138

[4] Cordova MJ Riba MB Spiegel D. Post-traumatic stress disorder and cancer. Lancet Psychiatry. (2017) 4:330–8. 10.1016/S2215-0366(17)30014-7 28109647 PMC5676567

[5] Wu X Wang J Cofie R Kaminga AC Liu A. Prevalence of posttraumatic stress disorder among breast cancer patients: a meta-analysis. Iran J Public Health. (2016) 45:1533–44. 10.1016/j.jpsychores.2020.109939 28053919 PMC5207094

[6] Vazquez D Rosenberg S Gelber S Ruddy KJ Morgan E Recklitis C. Posttraumatic stress in breast cancer survivors diagnosed at a young age. Psychooncology. (2020) 29:1312–20. 10.1002/pon.5438 32515073

[7] Kvillemo P Bränström R. Coping with breast cancer : a meta-analysis. PLoS ONE. (2014) 9:e112733. 10.1371/journal.pone.0112733 25423095 PMC4244095

[8] Arnaboldi P Riva S Crico C Pravettoni G. A systematic literature review exploring the prevalence of post-traumatic stress disorder and the role played by stress and traumatic stress in breast cancer diagnosis and trajectory. Breast Cancer. (2017) 9:473–85. 10.2147/BCTT.S111101 28740430 PMC5505536

[9] Zhao M Yang J Wang W Ma J Zhang J Zhao X. Meta-analysis of the interaction between serotonin transporter promoter variant, stress, and posttraumatic stress disorder. Sci Rep. (2017) 7:16532. 10.1038/s41598-017-15168-0 29184054 PMC5705670

[10] Kuzelova H Ptacek R Macek M. The serotonin transporter gene (5-HTT) variant and psychiatric disorders: review of current literature. Neuro Endocrinol Lett. (2010) 31:4–10. 20150867

[11] Gressier F Calati R Balestri M Marsano A Alberti S Antypa N. The 5-HTTLPR polymorphism and posttraumatic stress disorder: a meta-analysis. J Trauma Stress. (2013) 26:645–53. 10.1002/jts.21855 24222274

[12] Pietrzak RH Galea S Southwick SM Gelernter J. Examining the relation between the serotonin transporter 5-HTTPLR genotype x trauma exposure interaction on a contemporary phenotypic model of posttraumatic stress symptomatology: a pilot study. J Affect Disord. (2013) 148:123–8. 10.1016/j.jad.2012.11.003 23183127 PMC3604029

[13] Thakur GA Joober R Brunet A. Development and persistence of posttraumatic stress disorder and the 5-HTTLPR polymorphism. J Trauma Stress. (2009) 22:140–3. 10.1002/jts.20405 19444877

[14] Walsh K Uddin M Soliven R Wildman DE Bradley B. Associations between the SS variant of 5-HTTLPR and PTSD among adults with histories of childhood emotional abuse: results from two African American independent samples. J Affect Disord. (2014) 161:91–6. 10.1016/j.jad.2014.02.043 24751314 PMC4066731

[15] Grabe HJ Spitzer C Schwahn C Marcinek A Frahnow A Barnow S. Serotonin transporter gene (SLC6A4) promoter polymorphisms and the susceptibility to posttraumatic stress disorder in the general population. Am J Psychiatry. (2009) 166:926–33. 10.1176/appi.ajp.2009.08101542 19487392

[16] Sayin A Kucukyildirim S Akar T Bakkaloglu Z Demircan A Kurtoglu G. A prospective study of serotonin transporter gene promoter (5-HTT gene linked polymorphic region) and intron 2 (variable number of tandem repeats) polymorphisms as predictors of trauma response to mild physical injury. DNA Cell Biol. (2010) 29:71–7. 10.1089/dna.2009.0936 19895335

[17] Valente NLM Vallada H Cordeiro Q Miguita K Bressan RA Andreoli SB. Candidate-gene approach in posttraumatic stress disorder after urban violence: association analysis of the genes encoding serotonin transporter, dopamine transporter, and BDNF. J Mol Neurosci. (2011) 44:59–67. 10.1007/s12031-011-9513-7 21491204

[18] Navarro-Mateu F Escámez T Koenen KC Alonso J Sánchez-Meca J. Meta-analyses of the 5-HTTLPR polymorphisms and post-traumatic stress disorder. PLoS ONE. (2013) 25:e66227. 10.1371/journal.pone.0066227 23825531 PMC3692498

[19] Schillani G Martinis E Capozzo MA Era D Cristante T Mustacchi G. Psychological response to cancer: role of 5-HTTLPR genetic polymorphism of serotonin transporter. Anticancer Res. (2010) 30:3823–6. 20944177

[20] Gilbert J Haman KL Dietrich MS Blakely RD Shelton RC Murphy BA. Depression in patients with head and neck cancer and a functional genetic polymorphism of the serotonin transporter gene. Head Neck. (2012) 34:359–64. 10.1002/hed.21744 21604315

[21] Kim Y Carver CS Hallmayer JF Zeitzer JM Palesh O Neri E. Serotonin transporter polymorphism, depressive symptoms, and emotional impulsivity among advanced breast cancer patients. Support Care Cancer. (2018) 26:1181–8. 10.1007/s00520-017-3940-0 29090386 PMC7322579

[22] Bayer SJ Yang GS Lyon DE. Genetic variation associated with depressive symptoms in breast cancer patients: a systematic review. Cancer Nurs. (2020). 10.1097/NCC.0000000000000903. [Epub ahead of print]. 33156013

[23] Horowitz M Wilner N Alvarez W. Impact of event scale: a measure of subjective stress. Psychosom Med. (1979) 41:209–18. 10.1097/00006842-197905000-00004 472086

[24] Zilberg NJ Weiss DS Horowitz MJ. Impact of event scale: a cross-validation study and some empirical evidence supporting a conceptual model of stress response syndromes. J Consult Clin Psychol. (1982) 50:407–14. 10.1037/0022-006X.50.3.407 7096742

[25] Cella DF Mahon SM Donovan MI. Cancer recurrence as a traumatic event. Behav Med. (1990) 16:15–22. 10.1080/08964289.1990.9934587 2322653

[26] Sundin EC Horowitz MJ. Impact of Event Scale: psychometric properties. Br J Psychiatry. (2002) 180:205–9. 10.1192/bjp.180.3.205 11872511

[27] Voigt V Neufeld F Kaste J Bühner M Sckopke P Wuerstlein R. Clinically assessed posttraumatic stress in patients with breast cancer during the first year after diagnosis in the prospective, longitudinal, controlled COGNICARES study. Psychooncology. (2017) 26:74–80. 10.1002/pon.4102 26898732

[28] Salsman JM Schalet BD Andrykowski MA Cella D. The impact of events scale: a comparison of frequency versus severity approaches to measuring cancer-specific distress. Psychooncology. (2015) 24:1738–45. 10.1002/pon.3784 25773193 PMC4568176

[29] Paykel ES. The interview for recent life events. Psychol Med. (1997) 27:301–10. 10.1017/S0033291796004424 9089823

[30] Holland JC Bultz BD. The NCCN guideline for distress management: a case for making distress the sixth vital sign. J Natl Compr Canc Netw. (2007) 5:3–7. 10.6004/jnccn.2007.0003 17323529

[31] Riba MB Donovan KA Andersen B Braun I Breitbart WS Brewer BW. Distress Management, Version 3.2019, NCCN Clinical Practice Guidelines in Oncology. J. Natl. Compr. Canc. Netw. (2019) 17:1229–49. 10.6004/jnccn.2019.0048 31590149 PMC6907687

[32] Gelernter J Kranzler H Cubells JF. Serotonin transporter protein (SLC6A4) allele and haplotype frequencies and linkage disequilibria in African- and European-American and Japanese populations and in alcohol-dependent subjects. Hum Genet. (1997) 101:243–6. 10.1007/s004390050624 9402979

[33] Hayes AF. Introduction to Mediation, Moderation and Conditional Process Analysis - Appendices A and B (V3). New York, NY: Guilford Press (2017).

[34] Benjamini Y Hochberg Y. Controlling the false discovery rate - a practical and powerful approach to multiple testing. J R Stat Soc Ser B. (1995) 57:289–300. 10.1111/j.2517-6161.1995.tb02031.x

[35] Faul F Erdfelder E Lang AG Buchner A. G^*^Power 3: a flexible statistical power analysis program for the social, behavioral, and biomedical sciences. Behav Res Methods. (2007) 39:175–91. 10.3758/BF03193146 17695343

[36] Sharpley CF Palanisamy SKA Glyde NS Dillingham PW Agnew LL. An update on the interaction between the serotonin transporter promoter variant (5-HTTLPR), stress and depression, plus an exploration of non-confirming findings. Behav Brain Res. (2014) 273:89–105. 10.1016/j.bbr.2014.07.030 25078292

[37] Wang JS Conley YP Sereika SM Bender CM Godbole P Wesmiller SW. Examining the effect of 5-HTTLPR on depressive symptoms in postmenopausal women 1 year after initial breast cancer treatment. Support Care Cancer. (2019) 27:513–9. 10.1007/s00520-018-4332-9 29982901 PMC10049410

[38] Sola-Penna M Paixão LP Branco JR Ochioni AC Albanese JM Mundim DM. Serotonin activates glycolysis and mitochondria biogenesis in human breast cancer cells through activation of the Jak1/STAT3/ERK1/2 and adenylate cyclase/PKA, respectively. Br J Cancer. (2019) 122:194–208. 10.1038/s41416-019-0640-1 31819176 PMC7052254

[39] Zhu J Klein-Fedyshin M Stevenson JM. Serotonin transporter gene polymorphisms and selective serotonin reuptake inhibitor tolerability: review of pharmacogenetic evidence. Pharmacotherapy. (2017) 37:1089–104. 10.1002/phar.1978 28654193

[40] Suppli NP Bukh JD Moffitt TE Caspi A Johansen C Albieri V. 5-HTTLPR and use of antidepressants after colorectal cancer including a meta-analysis of 5-HTTLPR and depression after cancer. Transl Psychiatry. (2015) 5:e631. 10.1038/tp.2015.121 26327689 PMC5068816

[41] Breslau N Chilcoat HD Kessler RC Davis GC. Previous exposure to trauma and PTSD effects of subsequent trauma: results from the Detroit Area Survey of Trauma. Am J Psychiatry. (1999) 156:902–7. 10.1176/ajp.156.6.902 10360130

[42] Koenen KC Moffitt TE Poulton R Martin J Caspi A. Early childhood factors associated with the development of post-traumatic stress disorder: results from a longitudinal birth cohort. Psychol Med. (2007) 37:181–92. 10.1017/S0033291706009019 17052377 PMC2254221

[43] Roth MK Bingham B Shah A Joshi A Frazer A Strong R. Effects of chronic plus acute prolonged stress on measures of coping style, anxiety, and evoked HPA-axis reactivity. Neuropharmacology. (2012) 63:1118–26. 10.1016/j.neuropharm.2012.07.034 22842072 PMC3427462

[44] Weiss D. The impact of event scale: revised. In: Wilson JP Tang CS editors. Cross-Cultural Assessment of Psychological Trauma and PTSD. Boston, MA: Springer (2007). p. 219–38. 10.1007/978-0-387-70990-1_10

[45] Brown LC Candidate BS Murphy AR Lalonde CS. Posttraumatic stress disorder and breast cancer : risk factors and the role of inflammation and endocrine function. Cancer. (2020) 126:3181–91. 10.1002/cncr.32934 32374431 PMC9069707

[46] Dimitrov L Moschopoulou E Korszun A. Interventions for the treatment of cancer-related traumatic stress symptoms: a systematic review of the literature. Psychooncology. (2019) 28:970–9. 10.1002/pon.5055 30847978

[47] Haerizadeh M Sumner JA Birk JL Gonzalez C Heyman-Kantor R Falzon L. Interventions for posttraumatic stress disorder symptoms induced by medical events: a systematic review. J Psychosom Res. (2020) 129:109908. 10.1016/j.jpsychores.2019.109908 31884302 PMC7580195

