# Supplementary material for: Post-traumatic Stress Symptoms and Serotonin Transporter (5-HTTLPR) Polymorphism in Breast Cancer Patients
Source: Front Psychiatry. 2021 Apr 21;12:632596. doi: 10.3389/fpsyt.2021.632596 (PMC8097040; doi:10.3389/fpsyt.2021.632596)
Supplement: Supplementary file 1 [file Data_Sheet_1.docx]

**Table S1.** Sociodemographic, clinical and genotype characteristics of the sample

| **Age** (yrs) | 55.87 ± 8.98 (Range: 24-70) |
| --- | --- |
| **Education (yrs)** | 9.76 ± 4.34 (Range 5-18) |
| **Marital status** | |
| Never-married | 11 (7.6%) |
| Separated/divorced | 15(10.3%) |
| Married | 110 (75.8%) |
| Widow | 9 (6.2%) |
| **Stage** | |
| Local disease | 123 (84.8%) |
| Loco-regional | 22 (15.2%) |
| **Occupation** |  |
| Employed | 65 (44.8%) |
| Unemployed | 7 (4.8%) |
| Housewife | 29 (20%) |
| Retired | 44 (30.3%) |
| **Past psychological disorders** | |
| Yes | 50 (34.5%) |
| No | 95 (65.5%) |
| **Treatment** | |
| *Surgery* |  |
| Quadrantectomy | 93 (64.1%) |
| Conservative  (lumpectomy, sectoriectomy) | 9 (6.2%) |
| **Anticancer intervention** | |
| No Therapy | 70 (48.3%) |
| Chemotherapy | 33 (22.7%) |
| Hormone | 21 (14.5%) |
| Combined therapy (hormone+radio) | 21 (14.5%) |
| **5-HTTLPR** | |
| *l/l* | 45 patients (31.3%), |
| *s/l* | 66 *(*45.5%)*,* |
| *s/s* | 34 (23.4%) |
| (Hardy-Weinberg equilibrium: χ^2^=0.90416; p=ns | |

**Table S2.** Between-group differences in psychosocial variables according to 5-HTTLPR polymorphisms

|  | **Genotype** |  |  |  |  | **Phenotype** |  |  |  |
| --- | --- | --- | --- | --- | --- | --- | --- | --- | --- |
|  | ***s/s* (n=34)** | ***s/l* (n=66)** | ***l/l* (n=45)** | **Statistics** | ***p*** | ***s* allele**  **(n=100)** | **no *s* allele (n=45)** | **Statistics** | ***p*** |
| Age, mean ±SD | 55.1 ± 10.4 | 55.2 ± 8.8 | 56.1 ± 8.1 | F=0.13, df=2 | 0.80 | 55.7 | 56.1 | t=-0.26, df=1 | 0.8 |
| Localized stage | 30 | 58 | 35 | Χ^2^=3.68, df=2 | 0.15 | 88 | 35 | Χ^2^=3.36, df=1 | 0.06 |
| Loco-regional stage | 3 | 9 | 11 | Χ^2^=3.68, df=2 | 0.15 | 12 | 11 | Χ^2^=3.36, df=1 | 0.06 |
| No therapy | 17 | 29 | 25 | Χ^2^=1.48, df=2 | 0.48 | 46 | 25 | Χ^2^=0.88, df=1 | 0.35 |
| Chemotherapy | 6 | 18 | 14 | Χ^2^=1.54, df=2 | 0.46 | 24 | 14 | Χ^2^=0.68, df=1 | 0.41 |
| Radiotherapy | 4 | 12 | 4 | Χ^2^=2.04, df=2 | 0.36 | 16 | 4 | Χ^2^=1.42, df=1 | 2.33 |
| Hormonal therapy | 9 | 20 | 8 | Χ^2^=2.32, df=2 | 0.31 | 29 | 8 | Χ^2^=2.44, df=1 | 0.13 |
|  |  |  |  |  |  |  |  |  |  |
| Number of CRP, mean ±SD | 3.11 ± 3.18 | 4.1 ± 3.5 | 3.9 ± 3.4 | F=0.96, df=2 | 0.38 | 3.8 ± 3.5 | 3.9 ± 3.4 | t=0.04, df=144 | 0.80 |
| LSE, mean ±SD | 0.37 ± 0.54 | 0.37 ± 0.64 | 0.31 ± 0.68 | F=0.12, df=2 | 0.88 | 0.38 ± 0.61 | 0.31 ± 0.6 | t=0.48, df=144 | 0.46 |
| Number SLE | 11.4 ± 6.1 | 10.6 ± 6.8 | 11.6 ± 7.6 | F=0.32, df=2 | 0.72 | 10.8 ± 6.7 | 11.8 ± 7.5 | t=0.68, df=144 | 0.41 |
| Past psychiatric disorders | 9 | 23 | 20 | Χ^2^=2.29, df=2 | 0.31 | 32 | 20 | Χ^2^=1.17, df=1 | 0.17 |
|  |  |  |  |  |  |  |  |  |  |
| IES total score, mean ±SD |  |  |  |  |  |  |  |  |  |
| IES caseness (>35) | 7 | 9 | 10 | Χ^2^=1.62, df=2 | 0.44 | 16 | 10 | Χ^2^=0.71, df=1 | 0.41 |
| IES Intrusion, mean ±SD | 10.1 ± 8.8 | 7.61 ± 7.7 | 9.7 ± 9.4 | F=1.8, df=2 | 0.16 | 8.1 ± 8.1 | 9.7 ± 9.5 | t=1.2, df=144 | 0.20 |
| IES Avoidance, mean ±SD | 9.1 ±8.2 | 8.6 ± 8.3 | 10.7 ± 8.7 | F=0.87, df=2 | 0.46 | 8.8 ± 8.3 | 10.4 ± 8.7 | t=1.1, df=144 | 0.40 |

*Legend:* IES = Impact of Event Scale; MiniMAC/AP and H: Mini-Mental Adjustment to Cancer Scale Anxious Preoccupation and Hopelessness; MSPSS; Type D NA= Type D Negative affectivity; Type D SI = Type D Social Introversion; LSE = number of life stress events before diagnosis; CRP = cancer-related problems; SLE = other, non-cancer related, stressful life events

**Table S3.** Gene-environment interactions: sensitivity analysis

| **Outcome** | **Exposure** | **Test(s) of highest order**  **unconditional interactions(s):** | **Effects at levels of the moderator** | **Effect** | **se** | **p** | **LLCI** | **ULCI** |
| --- | --- | --- | --- | --- | --- | --- | --- | --- |
| **Cancer-related Problems** |  |  |  |  |  |  |  |  |
|  |  |  |  |  |  |  |  |  |
| **IES** | **CRP** | **R2-chng: 5%, F_2,142_=8.38, p=0.0044; q=**  **0.0167** | *S/S* | -0.68 | 0.57 | 0.23 | -1.82 | 0.45 |
|  |  |  | ***L/L*** | **2.43** | **0.91** | **0.00** | **0.63** | **4.23** |
|  |  |  |  |  |  |  |  |  |
| IES intrusion | CRP | R2-chng: 3%, F_2,140_=2.32, p=0.1020; q=  0.0500 | *S/S*  *S/L*  *L/L* | - | - | - | - | - |
| **IES avoidance** | **CRP** | **R2-chng 6%, F_2,140_=4.47, p=0.0131; q=**  **0.0333** | *S/S* | -0.47 | 0.51 | 0.35 | -1.49 | 0.53 |
|  |  |  | *S/L* | -0.64 | 0.41 | 0.11 | -1.45 | 0.16 |
|  |  |  | ***L/L*** | **1.20** | **0.50** | **0.01** | **0.20** | **2.19** |
|  |  |  |  |  |  |  |  |  |

R2-chng: change in explained variance by adding the moderating effect
